# Novel tumor marker index using carcinoembryonic antigen and carbohydrate antigen 19-9 is a significant prognostic factor for resectable colorectal cancer

**DOI:** 10.1038/s41598-024-54917-w

**Published:** 2024-02-20

**Authors:** Teppei Kamada, Hironori Ohdaira, Junji Takahashi, Takashi Aida, Keigo Nakashima, Eisaku Ito, Taigo Hata, Masashi Yoshida, Ken Eto, Yutaka Suzuki

**Affiliations:** 1 Department of Surgery, International University of Health and Welfare Hospital, 537-3, Iguchi, Nasushiobara, Tochigi 329-2763 Japan; 2 Department of Surgery, The Jikei University School of Medicine, 3-25-8, Nishi-shimbashi, Minato-ku, Tokyo, 105-8461 Japan

**Keywords:** Cancer, Gastroenterology, Oncology

## Abstract

We evaluated the usefulness of a newly devised tumor marker index (TMI), namely, the geometric mean of normalized carcinoembryonic antigen (CEA) and carbohydrate antigen 19-9 (CA19-9), in determining colorectal cancer (CRC) prognosis. This retrospective cohort study included 306 patients with stages I–III CRC who underwent elective laparoscopic resection between April 2010 and March 2020. Survival rates and risk factors of relapse-free survival (RFS) and cancer-specific survival (CSS) were analyzed using Kaplan–Meier curves and Cox proportional hazards model. High-TMI group (122 patients) had significantly lower rates (95% confidence interval [95% CI]) for 5-year RFS (89.7%, 83.9–93.5 vs. 65.8%, 56.3–73.8, p < 0.001) and CSS (94.9%, 89.4–97.6 vs. 77.3%, 67.7–84.4, p < 0.001) than low-TMI group. Multivariate analysis (hazard ratio [95% CI]) indicated ≥ T3 disease (RFS: 2.69, 1.12–6.45, p = 0.026; CSS: 7.64, 1.02–57.3, p = 0.048), stage III CRC (RFS: 3.30, 1.74–6.28, p < 0.001; CSS: 6.23, 2.04–19.0, p = 0.001), and high TMI (RFS: 2.50, 1.43–4.38, p = 0.001; CSS: 3.80, 1.63–8.87, p = 0.002) as significant RFS and CSS predictors. Area under the curve (AUC) of 5-year cancer deaths (0.739, p < 0.001) was significantly higher for TMI than for CEA or CA19-9 alone. Preoperative TMI is a useful prognostic indicator for patients with resectable CRC.

## Introduction

Colorectal cancer (CRC) ranks third in terms of incidence and is the second leading cause of death globally^1^. Radical resection is the recommended primary treatment for resectable CRC. In recent years, minimally invasive surgeries such as laparoscopic and robot-assisted surgery have become popular and are expected to improve the prognosis of CRC^2,3^. The recurrence rates for stages I and II CRC are 5.7% and 15.0%, respectively; however, that for stage III CRC is 31.8%, and this stage has a poor prognosis^4^. Therefore, early detection and identification of prognostic factors for CRC are essential. Recently, the usefulness of comprehensive genomic profiling (CGP) and circulating tumor DNA (ctDNA) using tumor or plasma samples has been reported. Furthermore, the early detection of cancer, identification of minimal residual tumor, and prediction of treatment effects are expected to have new clinical applications^5,6^. However, these methods are advanced medical approaches that require specialized equipment and are not widely used in clinical practice. Therefore, new biomarkers that can be detected easily and estimated with high accuracy are required. For example, tumor-related proteins secreted by cancerous tumors into the peripheral circulation could serve as biomarkers. Moreover, tumor markers are commonly examined in clinical practice as noninvasive indicators to diagnose cancer, evaluate tumor progression, and predict prognosis^7^. The usefulness of carcinoembryonic antigen (CEA), carbohydrate antigen 19-9 (CA19-9), carbohydrate antigen 242 (CA242), and tissue inhibitor of metalloproteinase-1 (TIMP-1) as tumor markers in CRC has been reported^8^. However, their diagnostic accuracy, including the sensitivity and specificity, are limited, and their prognostic impact remains low.

Recently, the value of tumor marker index (TMI) consisting of cytokeratin 19 fragment (CYFRA21-1) and squamous cell carcinoma-related antigen (SCC-Ag) as a prognostic factor in squamous esophageal cancer or CYFRA21-1 and CEA as a prognostic factor in non-small cell lung cancer has been reported^9,10^. However, the TMI has not been reported to be useful for CRC, in which adenocarcinoma is the main histological type.

In this study, we devised a novel TMI for CRC consisting of CEA and CA19-9, which are the most commonly used tumor markers in CRC. This study aimed to evaluate the usefulness of this novel TMI, in comparison with that of other existing prognostic factors, in determining the long-term prognosis of CRC.

## Methods

This retrospective cohort study included 306 patients who underwent elective laparoscopic surgery for CRC at our hospital, between April 2010 and March 2020.

The following patients were excluded: (1) those with pathological stage 0 or IV CRC, (2) those with multiple primary tumors, (3) those who underwent R1 or R2 resection, and (4) those who underwent preoperative chemoradiotherapy or lateral lymph node dissection for treating rectal cancer.

The primary endpoints were relapse-free survival (RFS) and cancer-specific survival (CSS). This study was approved by the Institutional Review Board of our hospital (approval no. 21-B-22), and the opt-out method was used to obtain informed consent. Briefly, details of the study procedures were provided on the website. Informed consent was considered to be obtained from all patients who did not opt out through the website.

Indications for surgery, selection of operation method or chemotherapy, and surveillance after curative resection for CRC were performed according to the clinical guidelines^4^. Pathological diagnoses were performed according to the Japanese classification of colorectal, appendiceal, and anal carcinoma^11^. Right-sided CRC was defined as a tumor located proximal to the splenic flexure, whereas left-sided CRC was defined as that located distal to the splenic flexure^12^. Postoperative complication was a Clavien–Dindo grade ≥ III complication occurring within 30 days after the surgery.

We collected the following data: age; sex; body mass index (BMI); tumor location, histopathology; tumor, node, metastasis classification; preoperative obstruction; surgical approach; surgical outcomes, including blood loss, operation time, postoperative complications, comorbidities, serum albumin (Alb) level, C-reactive protein (CRP) level, CEA level, and CA19-9 level, and leukocyte (neutrophil and lymphocyte) count.

### Establishment of the TMI in CRC

Blood tests were performed in all patients within a week before surgery. The cut-off values for CEA and CA19-9 were 5.0 ng/mL and 37.0 U/mL, respectively. TMI was defined as the geometric mean of the normalized values of serum CEA and CA19-9 levels. Normalization was performed by dividing the individual tumor marker values by the cut-off values. The TMI was calculated using a previously reported method^9^:

$$TMI=\sqrt{\frac{CEA\, (ng/mL)}{5.0}\times \frac{CA19{\text{-}}9\, (U/mL)}{37.0}}$$

The cut-off level of the TMI was determined by maximizing the Youden’s index for predicting 5-year cancer death; the optimal cut-off value of the TMI was 0.52. Using values above and below the cut-off values, the participants were categorized into high- and low-TMI groups, respectively.

### Measurement of other nutritional indexes

The Glasgow prognostic score (GPS), which is a combination of CRP and Alb, neutrophil-to-lymphocyte ratio (NLR), and platelet-to-lymphocyte ratio (PLR), was used as preoperative nutritional indexes, as previously reported^13,14^. The cut-off values of the NLR and PLR were 3.9 and 84, respectively, determined by maximizing the Youden’s index for predicting 5-year cancer death.

### Statistical analysis

To compare patient characteristics, categorical variables were analyzed using the Chi-square test, and continuous variables were analyzed using the Mann–Whitney *U* test. In survival analysis, Kaplan–Meier survival curves with log-rank test were used to estimate the survival function. Cox proportional hazards model was used to identify risk factors associated with worse RFS and CSS. Variables with a p-value < 0.05 according to the univariate analysis were subsequently included in the multivariate analysis, which involved a Cox proportional hazards model. The optimal cut-off values for continuous variables were defined as the values maximizing the Youden index for predicting 5-year survival on receiver-operating characteristic (ROC) curves. To assess the discriminative ability of the TMI, a ROC curve was plotted and the area under the curve (AUC) was computed. The AUC, shown as the absolute value and 95% confidence interval, provides a measure of the overall discriminatory ability of the TMI to predict 5-year survival.

The software program used to analyze the data was STATA/IC, version 16.0 (Stata Corp, College Station, TX, USA). A p-value < 0.05 was considered statistically significant.

### Ethics statements (humans ethics approval declaration)

The protocol for this research project has been approved by a suitably constituted ethics committee of the institution and it conforms to the provisions of the Declaration of Helsinki; the Institutional Review Board of the International University of Health and Welfare Hospital, approval no. 21-B-22.

### Patient consent statement

Informed consent was obtained from all the patients in the form of opt-out on the website.

## Results

### Comparison of baseline characteristics between groups

Table 1 shows the characteristics of the 306 patients who were classified into high-TMI (n = 122) and low-TMI groups (n = 184). This study included 192 males and 114 females. Patients in the high-TMI group had significantly lower BMI (p = 0.003), higher CEA and CA19-9 levels (p < 0.001 and p < 0.001, respectively), and more advanced T factor, N factor, and pathological staging than those in the low-TMI group (p < 0.001, p < 0.001, and p < 0.001, respectively). Furthermore, patients in the high-TMI group had longer operative duration (p = 0.005), more incidences of anastomotic leakage (p = 0.03) and ileus (p = 0.001), and received more adjuvant chemotherapy (p = 0.001) than those in the low-TMI group. No significant differences were observed between the two groups in terms of tumor location, histological type, operative procedure, degree of lymph node dissection, or preoperative nutritional status.

**Table 1:** Baseline characteristics in 306 patients who underwent laparoscopic colorectal resection: comparison between high-TMI and low-TMI groups.

Variables	Total	High-TMI	Low-TMI	p-value
		n (%) or median (range)		
Patients	306	122 (39.8%)	184 (60.2%)	
Age (years)	71.5 ± 11.0	72.1 ± 10.9	71.1 ± 11.1	0.41
Sex				
Male	192 (63%)	79 (64.7%)	113 (61.4%)	0.55
Female	114 (37%)	43 (35%)	71 (38.5%)	
Body mass index (kg/m^2^)	22.6 ± 4.12	21.8 ± 4.05	23.1 ± 4.09	0.003
Tumor location				0.06
Right-sided cancer	89 (29%)	28 (22.3%)	61 (33.1%)	
Left-sided cancer	217 (71%)	94 (77.0%)	123 (66.8%)	
Obstructive cancer	22 (7.2%)	13 (10.6%)	9 (4.9%)	0.06
CEA (ng/mL)	9.8 ± 24.8	19.8 ± 37.1	3.26 ± 3.43	< 0.001
CA19-9 (U/mL)	21.1 ± 43.9	42.6 ± 63.6	6.73 ± 5.22	< 0.001
Histopathology				0.20
tub1	147 (48%)	53 (43.4%)	94 (51.0%)	
tub2	147 (48%)	62 (50.8%)	85 (46.2%)	
por	4 (1.3%)	1 (0.8%)	3 (1.6%)	
Others	8 (2.7%)	6 (4.9%)	2 (1.1%)	
T factor				< 0.001
T1	63 (21%)	12 (9.8%)	51 (27.7%)	
T2	48 (16%)	16 (13.1%)	32 (17.3%)	
T3	173 (57%)	82 (67.2%)	91 (49.4%)	
T4	22 (7.2%)	12 (9.8%)	10 (5.4%)	
N factor				< 0.001
N0	189 (62%)	60 (49.2%)	129 (70.1%)	
N1	76 (25%)	34 (27.8%)	42 (22.8%)	
N2	36 (12%)	25 (20.4%)	11 (5.9%)	
N3	5 (1.6%)	3 (2.5%)	2 (1.1%)	
Pathological stage				< 0.001
I	92 (30.1%)	21 (17.2%)	71 (38.6%)	
II	97 (32%)	39 (31.9%)	58 (31.5%)	
III	117 (38%)	62 (50.8%)	55 (29.8%)	
Operative procedure				0.28
Ileocecal resection	40 (13%)	11 (9.0%)	29 (15.7%)	
Right hemicolectomy	45 (15%)	17 (13.9%)	28 (15.2%)	
Transverse colectomy	3 (1.0%)	0 (0%)	3 (1.9%)	
Left hemicolectomy	17 (5.6%)	6 (4.9%)	11 (5.9%)	
Sigmoid colectomy	99 (32%)	39 (31.9%)	60 (32.6%)	
Low anterior resection	67 (22%)	32 (26.2%)	35 (19.0%)	
Abdominoperineal resection	35 (11%)	17 (13.9%)	18 (9.8%)	
Lymph node dissection				0.37
D1	14 (4.6%)	6 (4.9%)	8 (4.4%)	
D2	117 (38%)	53 (43.4%)	64 (34.7%)	
D3	175 (57%)	63 (51.6%)	112 (60.8%)	
Operative time (min)	272.7 ± 78.4	289.2 ± 84.1	261.8 ± 72.5	0.005
Intraoperative blood loss (mL)	69.6 ± 150.8	83.3 ± 197.0	60.5 ± 109.8	0.70
Postoperative complication				
Anastomotic leakage	13 (4.3%)	9 (7.4%)	4 (2.1%)	0.03
Surgical site infection	47 (15%)	21 (17.2%)	26 (14.2%)	0.47
Ileus	37 (12%)	24 (19.6%)	13 (7.0%)	0.001
Intraperitoneal abscess	13 (4.3%)	8 (6.5%)	5 (2.7%)	0.10
Adjuvant chemotherapy	126 (41%)	64 (52.4%)	62 (33.7%)	0.001
High-NLR	46 (15%)	24 (19.6%)	22 (11.9%)	0.06
High-PLR	152 (49.6%)	60 (49.1%)	92 (50.0%)	0.88
GPS, 1 or 2	67 (21.9%)	33 (27.0%)	34 (18.5%)	0.08
Smoking history	109 (35.6%)	45 (36.8%)	64 (34.8%)	0.70
Liver cirrhosis	9 (2.9%)	3 (2.4%)	6 (3.3%)	0.68
Diabetes mellitus	59 (19.2%)	25 (20.5%)	34 (18.5%)	0.66

*CEA* carcinoembryonic antigen, *CA19-9* carbohydrate antigen 19-9, *tub1* well-differentiated tubular adenocarcinoma, *tub2* moderately differentiated tubular adenocarcinoma, *por* poorly differentiated adenocarcinoma, *NLR* neutrophil/lymphocyte ratio, *PLR* platelet-to-lymphocyte ratio, *GPS* Glasgow prognostic score, *TMI* tumor marker index.

The median follow-up period was 51.9 (range: 3.6–115.2) months. There were 58 (18.9%) relapse cases, 33 (10.7%) cancer deaths, and 53 (17.3%) deaths during the follow-up period. In this cohort, the 5-year RFS rate was 80.3% (95% confidence interval [95% CI]: 75.1–84.5), and the 5-year CSS rate was 87.9% (95% CI: 83.0–91.4).

### Risk factors for relapse after surgery for CRC

The associations between the clinicopathological variables and RFS are listed in Table 2. According to the univariate analysis, obstructive cancer (p < 0.01), CEA level ≥ 5.0 ng/mL (p < 0.01), CA19-9 level ≥ 37.0 ng/mL (p < 0.01), T3 or higher disease (p < 0.01), stage III CRC (p < 0.01), lymphovascular invasion (p < 0.01), and high TMI (p < 0.01) were potential risk factors for relapse. According to the multivariate analysis, T3 or higher disease (hazard ratio [HR]: 2.69, 95% CI: 1.12–6.45, p = 0.026), stage III CRC (HR: 3.30, 95% CI: 1.74–6.28, p < 0.001), and high TMI (HR: 2.50, 95% CI: 1.43–4.38, p = 0.001) were independent risk factors for relapse.

**Table 2:** Univariate and multivariate analyses of relapse-free survival in patients with colorectal cancer after surgery.

Variables	Univariate		Multivariate	
	HR (95% CI)	p-value	HR (95% CI)	p-value
Age, ≥ 65 years	1.78 (0.87–3.61)	0.11		
Sex, male	1.40 (0.80–2.45)	0.23		
Right-sided cancer	0.92 (0.52–1.63)	0.77		
Obstructive colorectal cancer	2.70 (1.32–5.51)	< 0.01	1.38 (0.67–2.86)	0.37
CEA, ≥ 5.0 ng/mL	3.15 (1.87–5.30)	< 0.01		
CA19-9, ≥ 37.0 U/mL	3.15 (1.73–5.76)	< 0.01		
T factor, T3 or higher	5.75 (2.46–13.4)	< 0.01	2.69 (1.12–6.45)	0.026
Stage III	6.06 (3.32–11.1)	< 0.01	3.30 (1.74–6.28)	< 0.001
Lymphovascular invasion, positive	7.19 (2.25–23.0)	< 0.01	2.57 (0.75–8.73)	0.13
Complications, including leakage or ileus	1.50 (0.65–3.50)	0.34		
GPS, 1 or 2	1.29 (0.71–2.36)	0.40		
High-NLR	1.56 (0.81–3.02)	0.18		
High-PLR	1.16 (0.69–1.94)	0.57		
High-TMI	3.70 (2.13–6.41)	< 0.01	2.51 (1.43–4.38)	0.001

*HR* hazard ratio, *CI* confidence interval, *CEA* carcinoembryonic antigen, *CA19-9* carbohydrate antigen 19-9, *GPS* Glasgow Prognostic Score, *NLR* neutrophil-to-lymphocyte ratio, *PLR* platelet-to-lymphocyte ratio, *TMI* tumor marker index.

### Risk factors for cancer-specific mortality after surgery for CRC

The associations between the clinicopathological variables and CSS are listed in Table 3. According to the univariate analysis, obstructive cancer (p = 0.045), CEA level ≥ 5.0 ng/mL (p < 0.01), CA19-9 level ≥ 37.0 ng/mL (p < 0.01), T3 or higher disease (p < 0.01), stage III CRC (p < 0.01), lymphovascular invasion (p = 0.019), and high TMI (p < 0.01) were potential risk factors for cancer-specific mortality. According to the multivariate analysis, T3 or higher disease (HR: 7.64, 95% CI: 1.02–57.3, p = 0.048), stage III CRC (HR: 6.23, 95% CI: 2.04–19.0, p = 0.001), and high TMI (HR: 3.80, 95% CI: 1.63–8.87, p = 0.002) were independent risk factors for cancer-specific mortality.

**Table 3:** Univariate and multivariate analyses of cancer-specific survival in patients with colorectal cancer after surgery.

Variables	Univariate		Multivariate	
	HR (95% CI)	p-value	HR (95% CI)	p-value
Age, ≥ 65 years	1.33 (0.58–3.09)	0.49		
Sex, male	2.09 (0.94–4.65)	0.07		
Right-sided cancer	0.96 (0.44–2.07)	0.92		
Obstructive colorectal cancer	2.65 (1.02–6.89)	0.045	1.13 (0.43–2.98)	0.79
CEA, ≥ 5.0 ng/mL	3.07 (1.53–6.12)	< 0.01		
CA19-9, ≥ 37.0 U/mL	4.89 (2.40–9.95)	< 0.01		
T factor, T3 or higher	19.6 (2.68–143.9)	< 0.01	7.64 (1.01–57.3)	0.048
Stage III	11.8 (4.16–33.7)	< 0.01	6.23 (2.04–19.0)	0.001
Lymphovascular invasion, positive	5.52 (1.32–23.0)	0.019	1.25 (0.27–5.71)	0.76
Complications, including leakage or ileus	1.48 (0.45–4.87)	0.51		
GPS, 1 or 2	1.41 (0.63–3.13)	0.39		
High NLR	1.61 (0.66–3.91)	0.29		
High-PLR	0.80 (0.40–1.61)	0.54		
High-TMI	6.12 (2.65–14.1)	< 0.01	3.80 (1.63–8.87)	0.002

*HR* hazard ratio, *CI* confidence interval, *CEA* carcinoembryonic antigen, *CA19-9* carbohydrate antigen 19-9, *GPS* Glasgow Prognostic Score, *NLR* neutrophil-to-lymphocyte ratio, *PLR* platelet-to-lymphocyte ratio, *TMI* tumor marker index.

### RFS and CSS analysis

**Figure 1:**
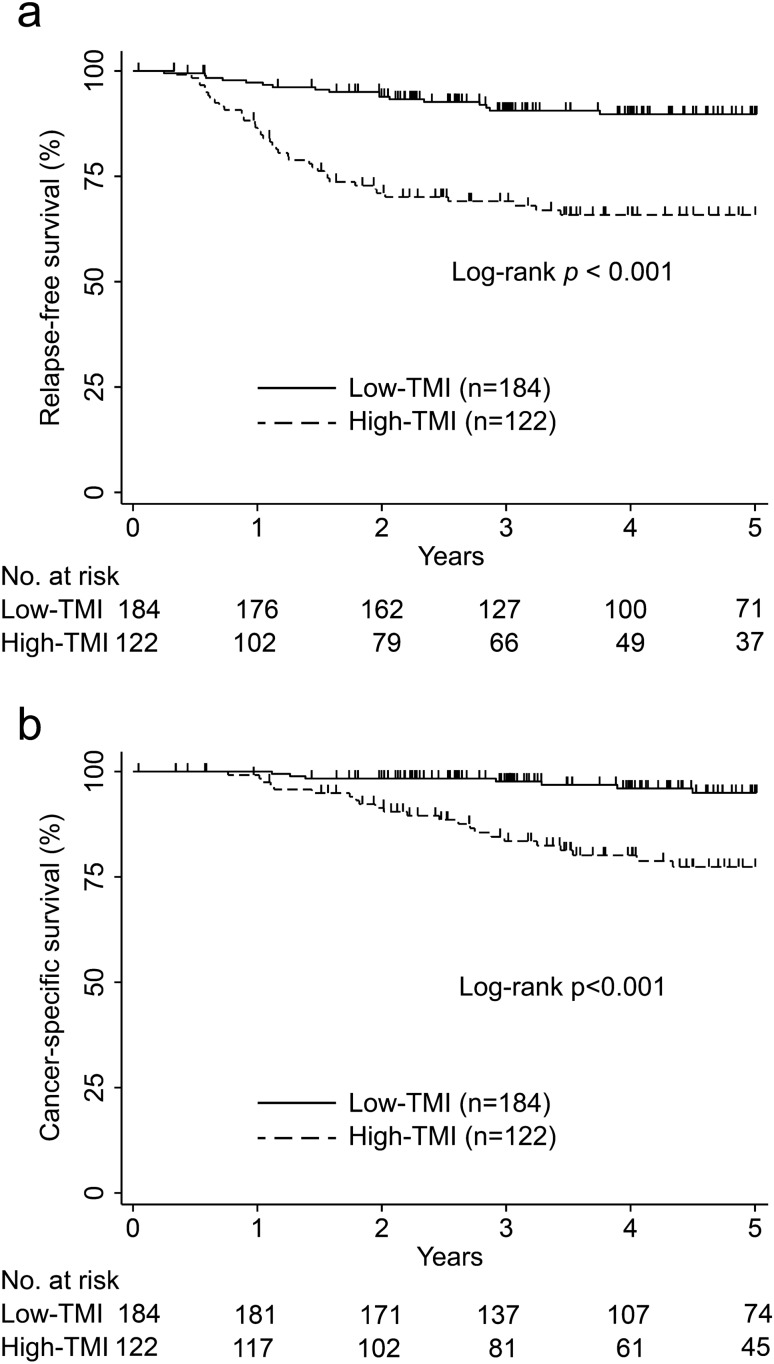
Kaplan–Meier curve of survival after surgery in patients with stages I–III colorectal cancer evaluated using tumor marker index levels. (**a**) Relapse-free survival and (**b**) cancer-specific survival.

Kaplan–Meier survival curves in the high- and low-TMI groups are presented in Fig. 1. Figure 1a demonstrates that patients with high TMI had significantly lower RFS rates than those with low TMI (5-year RFS rates: 89.7%, 95% CI: 83.9–93.5 vs. 65.8%, 95% CI: 56.3–73.8; p < 0.001; Fig. 1a). Furthermore, Fig. 1b demonstrates that patients with high TMI had significantly lower CSS rates than those with low TMI (5-year CSS rates: 94.9%, 95% CI: 89.4–97.6 vs. 77.3%, 95% CI: 67.7–84.4; p < 0.001; Fig. 1b).

### Subgroup analysis

With regard to patient characteristics (Table 1), the high-TMI group had significantly advanced T-factor, N-factor, and advanced pathological staging. Consequently, for subgroup analysis, the patients were classified into three subgroups based on pathological staging: (1) stage I (n = 92), (2) stage II (n = 97), (3) stage III (n = 117).

#### RFS analysis

**Figure 2:**
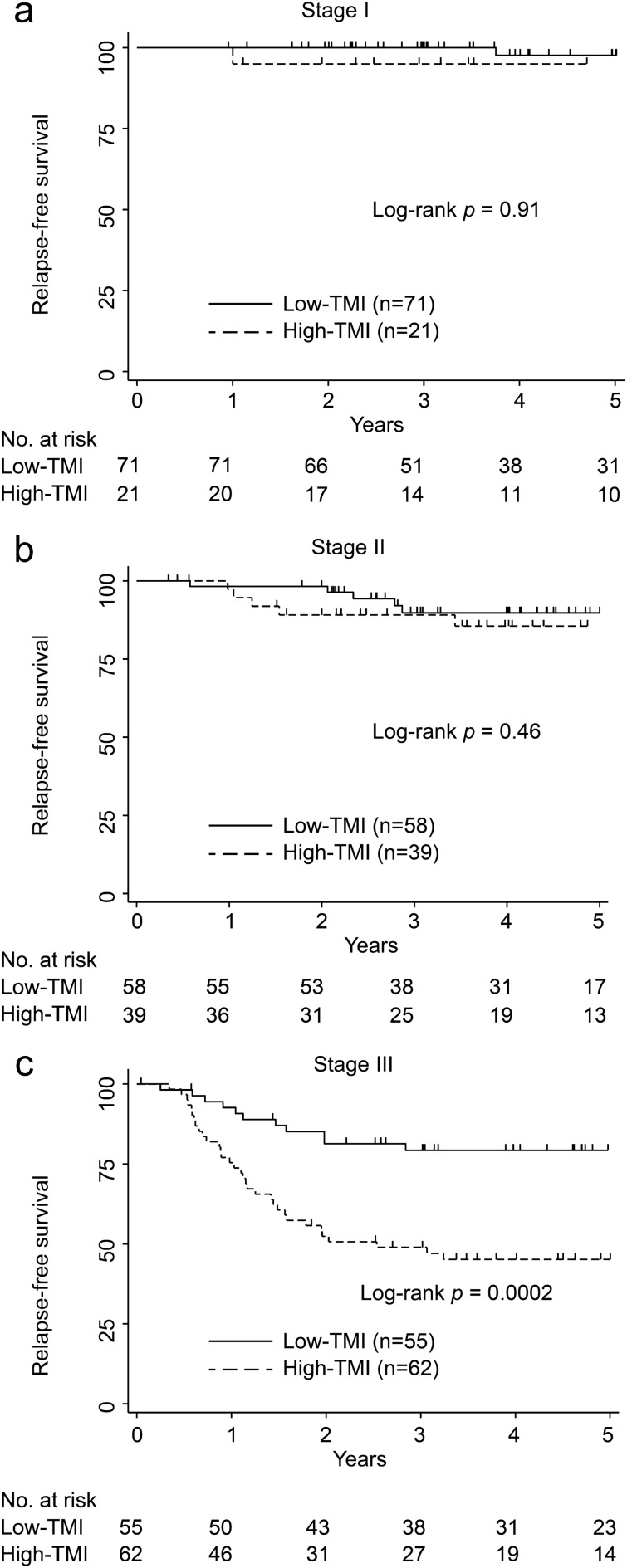
Kaplan–Meier curve of relapse-free survival after surgery in patients with stages I–III colorectal cancer evaluated using tumor marker index levels. (**a**) Stage I, (**b**) stage II, and (**c**) stage III colorectal cancer.

The comparisons of the Kaplan–Meier RFS curves in each stage are shown in Fig. 2a–c. In the stage I CRC group, no significant differences in the RFS rates were observed between the high- and low-TMI groups (5-year RFS rates: 97.6%, 95% CI: 84.3–99.6 vs. 95.0%, 95% CI: 69.4–99.3; p = 0.91; Fig. 2a). Furthermore, in the stage II CRC group, there were no significant differences in RFS rates between the high- and low-TMI groups (5-year RFS rates: 89.7%, 95% CI: 77.0–95.6 vs. 85.5%, 95% CI: 68.4–93.7; p = 0.46; Fig. 2b). In contrast, in the stage III CRC group, patients with high TMI had significantly lower RFS than those with low TMI (5-year RFS rates: 79.2%, 95% CI: 65.6–87.9 vs. 45.1%, 95% CI: 32.2–57.2; p = 0.0002; Fig. 2c).

#### CSS analysis

**Figure 3:**
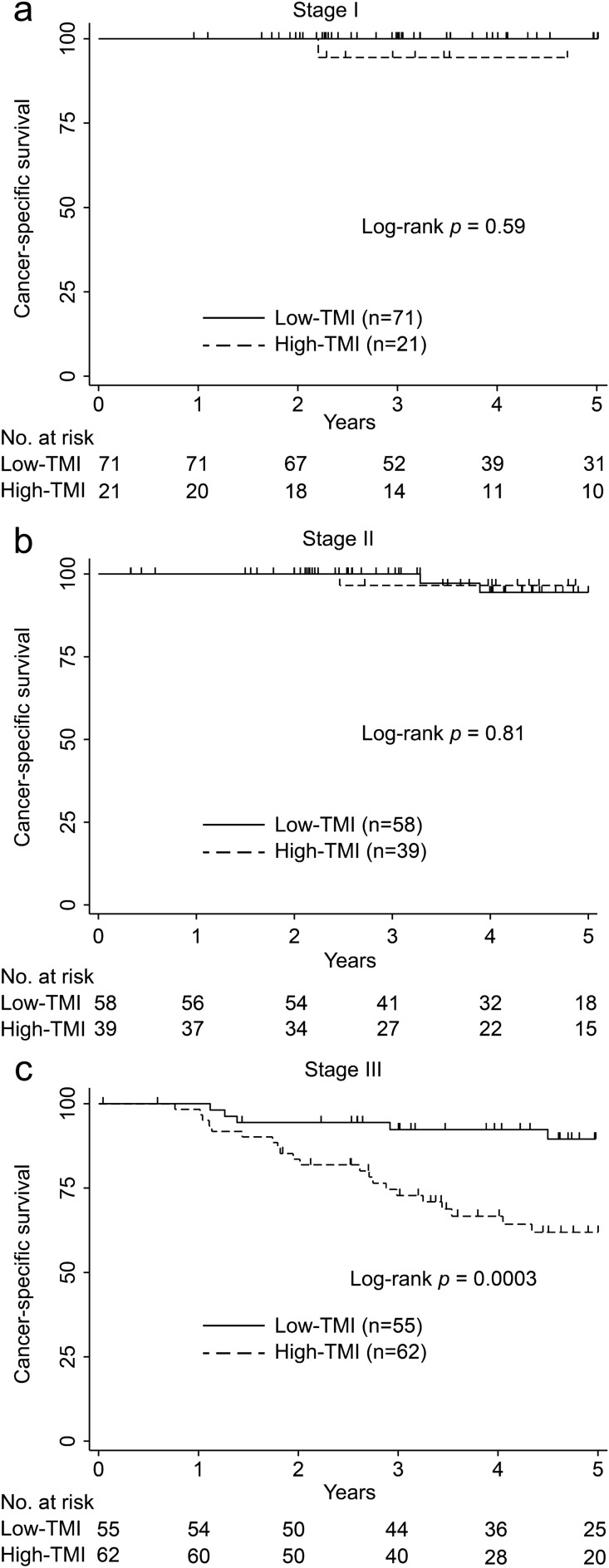
Kaplan–Meier curve of cancer-specific survival after surgery of patients with stages I–III colorectal cancer by tumor marker index levels. (**a**) Stage I, (**b**) stage II, and (**c**) stage III colorectal cancer.

The comparisons of the Kaplan–Meier CSS curves for each stage are shown in Fig. 3a–c. In the stage I CRC group, there were no significant differences in CSS rates between the high- and low-TMI groups (5-year CSS rates: 100% vs. 94.4%, 95% CI: 66.6–99.2; p = 0.059; Fig. 3a). In the stage II CRC group, there were no significant differences in CSS rates between the high- and low-TMI groups (5-year CSS rates: 94.4%, 95% CI: 79.5–98.5 vs. 96.5%, 95% CI: 77.9–99.5; p = 0.81; Fig. 3b). In contrast, in the stage III CRC group, patients with high TMI had significantly lower CSS than those with low TMI (5-year CSS rates: 89.5%, 95% CI: 76.3–95.5 vs. 61.9%, 95% CI: 47.3–73.5; p = 0.0003; Fig. 3c).

### Comparison of the survival curves between patients with or without elevated CEA and CA19-9 levels

**Figure 4:**
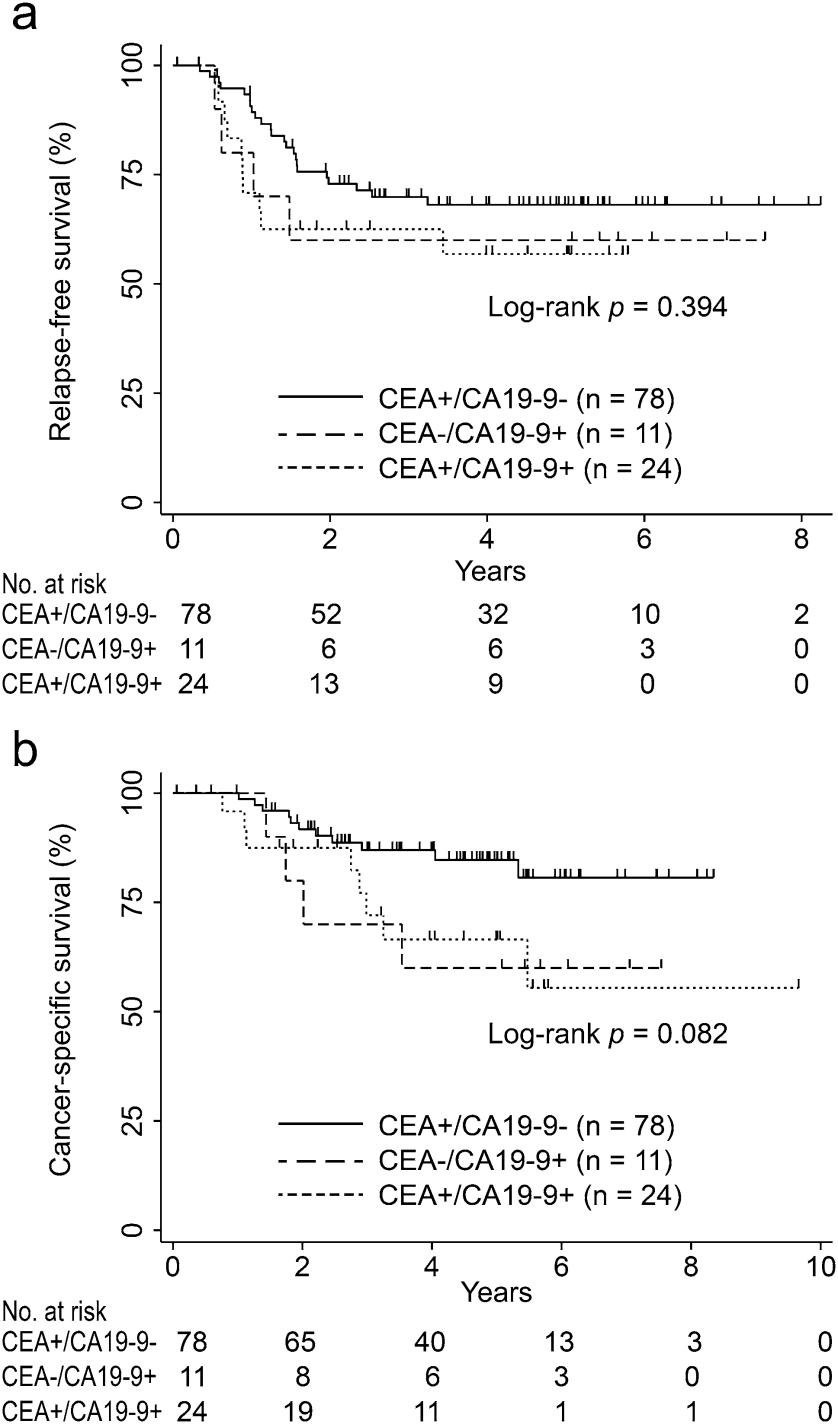
Kaplan–Meier curve of survival after surgery in patients with stage I–III colorectal cancer according to carcinoembryonic antigen and carbohydrate antigen 19-9 levels. (**a**) Relapse-free survival and (**b**) cancer-specific survival.

In addition, the patients were classified into three subgroups based on the levels of CEA and CA 19-9 to determine the RFS and CSS: (1) increase in CEA levels only (CEA+/CA19-9−), (2) increase in CA 19-9 levels only (CEA−/CA19-9+), and (3) increase in CEA and CA19-9 levels (CEA+/CA19-9+). There were no significant differences in RFS rates among the three groups (5-year RFS rates: [CEA+/CA19-9−]: 68.1%, 95% CI: 55.8–77.5 vs. [CEA−/CA19-9+]: 60.0%, 95% CI: 25.3–82.7 vs. [CEA+/CA19-9+]: 56.8%, 95% CI: 34.2–74.2; p = 0.39; Fig. 4a). Furthermore, there were no significant differences in CSS rates among the three groups (5-year CSS rates: [CEA+/CA19-9−]: 84.7%, 95% CI: 73.1–91.5 vs. [CEA−/CA19-9+]: 60.0%, 95% CI: 25.3–82.7 vs. [CEA+/CA19-9+]: 66.5%, 95% CI: 41.8–82.6; p = 0.082; Fig. 4b).

### ROC curves for the TMI, CEA level, and CA19-9 level for predicting 5-year survivals after colorectal surgery

**Figure 5:**
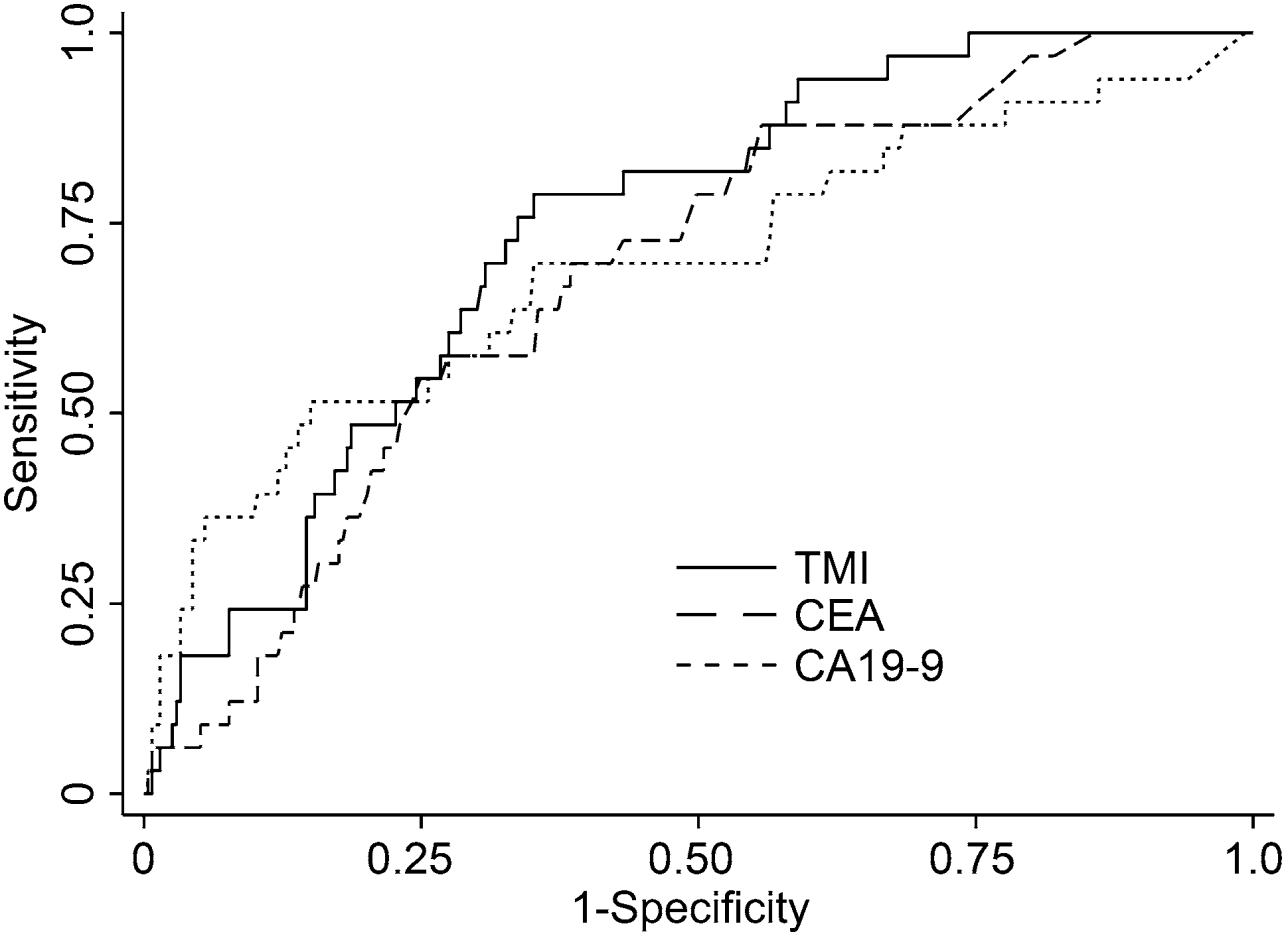
ROC curves of the prediction of 5-year survival comparing TMI, CEA, and CA 19-9 levels of patients with CRC. *CA* carbohydrate antigen, *CEA* carcinoembryonic antigen, *ROC* receiver operating characteristic, *TMI* tumor marker index.

The diagnostic accuracy of the TMI and existing tumor markers, including CEA and CA19-9 levels, were compared using ROC curves. Figure 5 demonstrates that the AUC of the TMI for 5-year survival was significantly higher (TMI: 0.739, 95% CI: 0.66–0.81; p < 0.001) compared to that of CEA or CA19-9 alone; (CEA: 0.682, 95% CI: 0.59–0.76; CA19-9: 0.695, 95% CI: 0.58–0.80, respectively).

## Discussion and conclusions

This study suggested that TMI comprising preoperative serum CEA and CA19-9 levels could be a useful prognostic factor for determining RFS and CSS in patients with CRC. The results of subgroup analysis also showed that this TMI was a useful prognostic predictor, especially in stage III CRC. To the best of our knowledge, this is the first study to demonstrate the effectiveness of our newly devised TMI in determining the long-term prognosis of CRC.

CEA is a representative tumor marker discovered in CRC tissue; it is a glycoprotein normally found in the embryonic endodermal epithelium expressed in many epithelial tumors^15^. It was discovered by Gold et al. in 1965 and is the most recommended marker for postoperative surveillance of CRC^8,15,16^. CEA is widely used not only for surveillance following curative resection but also for determining prognosis or monitoring therapy in patients with advanced CRC^8^.

In contrast, CA19-9, which was discovered by Koprowski in 1979, is a mouse monoclonal antibody NS19-9 raised against the human colonic carcinoma cell line SW1116^17^. CA19-9 and CEA are widely used to determine the prognosis and monitor therapeutic effects in CRC^8,18^. The measurement of these tumor markers is less expensive and simple, but the diagnostic accuracy of each tumor marker is limited. The sensitivity and specificity of CEA are 64.5 and 89.2%, respectively, whereas those of CA19-9 are 47.8 and 90.1%, respectively^19,20^. Several studies combine two or more biomarkers to effectively improve the accuracy and efficiency of the biomarkers in CRC diagnosis^19,21,22^. Although the combination of CEA and CA19-9 has been reported to show high diagnostic accuracy for CRC, few reports describe the use of a combination of both CEA and CA19-9 together as a prognostic predictor of CRC.

In this study, we performed a subgroup analysis of the three groups (stages I–III CRC) using cut-off values of CEA and CA19-9 (CEA: 5.0 ng/mL, CA19-9: 37.0 U/mL), which are frequently used in clinical practice. There were no significant differences in RFS or CSS among the three groups (p = 0.39 and p = 0.082, respectively) (Fig. 4). The results indicated that it is difficult to identify the groups with poor prognosis using the cut-off values of CEA and CA19-9 in clinical practice.

Therefore, in order to use the potentially synergistic effect of the combination of CEA and CA19-9 to predict CRC prognosis, a TMI based on CEA and CA19-9 was designed as an independent biomarker. Nanchang et al. state that TMIs based on CYFRA21-1 and SCC-Ag could be useful prognostic indicators in patients with esophageal squamous cell carcinoma undergoing radical resection^9^. However, as a limitation of previous studies, the T-factor, N-factor, and pathological stage were significantly more advanced in the high-TMI group than in the low-TMI group, and no subgroup analysis for each stage was performed in these studies. Therefore, to unify the tumor staging, we performed subgroup analysis for stages I–III CRC and found that the prognosis in patients with high TMI was significantly worse in terms of RFS and CSS than that in patients with low TMI, particularly in those with stage III CRC.

We attribute the lack of significant differences in RFS and CSS between the high-TMI and low-TMI groups between stages I and II to the following reasons: (1) the number of patients was too small (stage I: n = 92, stage II: n = 97) and (2) the numbers of events of relapse and cancer-death were too small due to the high prevalence of early-stage disease. In addition, the AUC of the TMI for 5-year cancer-death rate was significantly higher than that of either CEA or CA19-9 alone, indicating that TMI is a more sensitive prognostic predictor than either of these factors alone.

The TMI designed herein has several advantages. First, it can be calculated using a simple formula with the existing tumor markers and is easy to apply clinically because it is an independent indicator with a cut-off value. Second, TMI measurement is a noninvasive procedure and does not incur additional medical costs. Therefore, our TMI may be a useful prognostic biomarker of CRC.

Another interesting finding of this study was that the high-TMI group had significantly longer operative durations and significantly more postoperative complications, including anastomotic leakage or ileus, than the low-TMI group. The possible reason for the higher number of complications in the high-TMI group is that there were more significantly advanced T-factors and N-factors in the high-TMI group, which made the surgery more invasive, resulting in more short-term complications, such as anastomotic leakage or ileus. This suggests that the TMI may be a useful biomarker as a predictor of both long-term prognosis and short-term outcomes.

One limitation of this study is its small sample size, retrospective nature, and single-center cohort design. Another potential limitation is that not all confounding factors were analyzed. Furthermore, because TMI is a newly devised index with no clear cut-off value, it is necessary to determine a universal cut-off value, confirm the usefulness of the TMI formula using large-scale data, and verify the usefulness with a validation cohort in the future.

In conclusion, our study suggested that a high preoperative TMI is a useful long-term prognostic indicator in stages I–III resectable CRC. The use of the TMI combining CEA and CA19-9 is expected to lead to the early identification of patients with CRC with poor prognosis, which may improve survival in this patient population.

## Data Availability

The data that support the findings of this study are available from the corresponding author upon reasonable request.

## References

[1] Sung H (2021). Global cancer statistics 2020: GLOBOCAN estimates of incidence and mortality worldwide for 36 cancers in 185 countries. CA Cancer J. Clin..

[2] Safiejko K (2021). Robotic-assisted vs. standard laparoscopic surgery for rectal cancer resection: A systematic review and meta-analysis of 19,731 patients. Cancers (Basel).

[3] Yuval JB (2023). Comparison of robotic, laparoscopic, and open resections of nonmetastatic colon cancer. Dis. Colon Rectum.

[4] Hashiguchi Y (2020). Japanese society for cancer of the colon and rectum (JSCCR) guidelines 2019 for the treatment of colorectal cancer. Int. J. Clin. Oncol..

[5] Malla M, Loree JM, Kasi PM, Parikh AR (2022). Using circulating tumor DNA in colorectal cancer: Current and evolving practices. J. Clin. Oncol..

[6] Mo S (2023). Early detection of molecular residual disease and risk stratification for stage I to III colorectal cancer via circulating tumor DNA methylation. JAMA Oncol..

[7] Yamashita K, Watanabe M (2009). Clinical significance of tumor markers and an emerging perspective on colorectal cancer. Cancer Sci..

[8] Duffy MJ (2007). Tumour markers in colorectal cancer: European Group on Tumour Markers (EGTM) guidelines for clinical use. Eur. J. Cancer.

[9] Yin N, Liu W (2020). Clinical value of tumor marker index based on preoperative CYFRA 21-1 and SCC-Ag in the evaluation of prognosis and treatment effectiveness in patients with esophageal squamous cell carcinoma. Onco Targets Ther..

[10] Muley T (2008). Tumor volume and tumor marker index based on CYFRA 21-1 and CEA are strong prognostic factors in operated early stage NSCLC. Lung Cancer.

[11] Japanese Society for Cancer of the Colon and Rectum (2019). Japanese Classification of Colorectal, Appendiceal, and Anal Carcinoma: The 3d English Edition [Secondary Publication]. J. Anus Rectum Colon.

[12] Belias M (2022). Is laterality prognostic in resected KRAS-mutated colorectal liver metastases? A systematic review and meta-analysis. Cancers (Basel).

[13] Forrest LM, McMillan DC, McArdle CS, Angerson WJ, Dunlop DJ (2003). Evaluation of cumulative prognostic scores based on the systemic inflammatory response in patients with inoperable non-small-cell lung cancer. Br. J. Cancer.

[14] Yamamoto T, Kawada K, Obama K (2021). Inflammation-related biomarkers for the prediction of prognosis in colorectal cancer patients. Int. J. Mol. Sci..

[15] Quentmeier A, Möller P, Schwarz V, Abel U, Schlag P (1987). Carcinoembryonic antigen, CA 19.9, and CA 125 in normal and carcinomatous human colorectal tissue. Cancer.

[16] Gold P, Freedman SO (1965). Demonstration of tumor-specific antigens in human colonic carcinomata by immunological tolerance and absorption techniques. J. Exp. Med..

[17] Koprowski H (1979). Colorectal carcinoma antigens detected by hybridoma antibodies. Somatic Cell Genet..

[18] Duffy MJ (2014). Tumor markers in colorectal cancer, gastric cancer and gastrointestinal stromal cancers: European group on tumor markers 2014 guidelines update. Int. J. Cancer.

[19] Zhang SY, Lin M, Zhang HB (2015). Diagnostic value of carcinoembryonic antigen and carcinoma antigen 19-9 for colorectal carcinoma. Int. J. Clin. Exp. Pathol..

[20] Jelks W, Mroczko B (2020). Biochemical markers of colorectal cancer—Present and future. Cancer Manag. Res..

[21] Zhou H, Wang XC, Yuan BB, Lu BB (2022). Clinical value of combining serum tumor marker detection with fecal occult blood testing in diagnosing colorectal cancer. J. Physiol. Pharmacol..

[22] Stiksma J, Grootendorst DC, van der Linden PW (2014). CA 19-9 as a marker in addition to CEA to monitor colorectal cancer. Clin. Colorectal Cancer.

